# Buffy coat signatures of breast cancer risk in a prospective cohort study

**DOI:** 10.1186/s13148-023-01509-6

**Published:** 2023-06-12

**Authors:** Felicia Fei-Lei Chung, Sandra González Maldonado, Amelie Nemc, Liacine Bouaoun, Vincent Cahais, Cyrille Cuenin, Aurelie Salle, Theron Johnson, Bekir Ergüner, Marina Laplana, Paul Datlinger, Jana Jeschke, Elisabete Weiderpass, Vessela Kristensen, Suzette Delaloge, François Fuks, Angela Risch, Akram Ghantous, Christoph Plass, Christoph Bock, Rudolf Kaaks, Zdenko Herceg

**Affiliations:** 1 International Agency for Research On Cancer (IARC), 25 avenue Tony Garnier, CS 90627, 69366 Lyon, France; 2 Department of Medical Sciences, School of Medical and Life Sciences, Sunway University, 5, Jalan Universiti, Bandar Sunway, 47500 Subang Jaya, Selangor Malaysia; 3 Division of Cancer Epidemiology, German Cancer Research Center (DKFZ), Heidelberg, Germany; 4 CeMM Research Center for Molecular Medicine of the Austrian Academy of Sciences, Vienna, Austria; 5 Division of Cancer Epigenomics, German Cancer Research Center, Heidelberg, Germany; 6 Department of Basic Medical Sciences, University of Lleida, IRBLleida, 25198 Lleida, Spain; 7 Laboratory of Cancer Epigenetics, Université Libre de Bruxelles (ULB), Brussels, Belgium; 8 Faculty of Medicine, Institute for Clinical Epidemiology and Molecular Biology, University of Oslo, Oslo, Norway; 9 Department of Cancer Medicine, Institut Gustave Roussy, Villejuif, France; 10 Department of Biosciences and Medical Biology, Allergy-Cancer-BioNano Research Centre, University of Salzburg, 5020 Salzburg, Austria; 11 Cancer Cluster Salzburg, Salzburg, Austria; 12 Medical University of Vienna, Institute of Artificial Intelligence, Center for Medical Data Science, Vienna, Austria

**Keywords:** Epigenetics, DNA methylation, Cancer risk markers, Breast cancer, Prospective cohort

## Abstract

**Background:**

Epigenetic alterations are a near-universal feature of human malignancy and have been detected in malignant cells as well as in easily accessible specimens such as blood and urine. These findings offer promising applications in cancer detection, subtyping, and treatment monitoring. However, much of the current evidence is based on findings in retrospective studies and may reflect epigenetic patterns that have already been influenced by the onset of the disease.

**Methods:**

Studying breast cancer, we established genome-scale DNA methylation profiles of prospectively collected buffy coat samples (*n* = 702) from a case–control study nested within the EPIC-Heidelberg cohort using reduced representation bisulphite sequencing (RRBS).

**Results:**

We observed cancer-specific DNA methylation events in buffy coat samples. Increased DNA methylation in genomic regions associated with SURF6 and REXO1/CTB31O20.3 was linked to the length of time to diagnosis in the prospectively collected buffy coat DNA from individuals who subsequently developed breast cancer. Using machine learning methods, we piloted a DNA methylation-based classifier that predicted case–control status in a held-out validation set with 76.5% accuracy, in some cases up to 15 years before clinical diagnosis of the disease.

**Conclusions:**

Taken together, our findings suggest a model of gradual accumulation of cancer-associated DNA methylation patterns in peripheral blood, which may be detected long before clinical manifestation of cancer. Such changes may provide useful markers for risk stratification and, ultimately, personalized cancer prevention.

**Supplementary Information:**

The online version contains supplementary material available at 10.1186/s13148-023-01509-6.

## Background

Cancer is a leading cause of death worldwide and has been described as the single most important barrier to increasing life expectancy in the twenty-first century [1]. While the development of effective screening procedures has allowed for early detection of malignant lesions and reductions in cancer-related mortality [2], few early detection tests have been effective in reducing cancer-specific morbidity to date [3]. There is a need to re-examine the limitations of the current “one-size-fits-all” approach to cancer screening and to move towards more personalized approaches for prevention and early detection [4].

One strategy towards addressing this challenge is to integrate molecular markers in the generation of risk stratification profiles [2, 4]. Epigenetic markers have been put forward as important indicators of cancer risk, and they are highly attractive options in clinical practice because of their technical stability [4, 5]. Epigenetic measures of biological age, in particular, have been associated with cancer-related mortality [6–11] and have great potential utility as early biomarkers of disease risk [12]. Multiple studies to date have established that alterations in DNA methylation can be detected in DNA isolated from the peripheral blood of patients with cancer [13–17]. Recent reports combining epigenomic analyses with machine learning classifiers were able to infer not only the presence of tumours but also the tissue of origin or subtype of the tumours [18–23]. Although these findings offer promising evidence for the utility of epigenetic events as biomarkers or predictors of cancer, these studies are retrospective in nature, reporting on methylation markers that are detectable upon or after diagnosis.

To add value as an early detection or risk stratification strategy, proposed assays should be non-invasive and capable of detecting cellular alterations before the disease progresses to the lower detection limit of conventional screening modalities. To date, reports indicate that epigenetic markers can be detected in prospectively collected from apparently healthy individuals that are later diagnosed with breast [24, 25] and ovarian cancers [26], suggesting that the DNA methylation profile in peripheral blood may be altered years before the tumour is clinically detected. A report from the Taizhou Longitudinal Study revealed that an epigenomics-based blood test could identify stomach, oesophageal, colorectal, lung, or liver cancer in apparently healthy individuals up to 4 years before diagnosis [27]. However, separate meta-analyses on similar pre-diagnostic samples reported no associations between risk of breast cancer [28] or gastric cancer [29] and DNA methylation measured at individual CpG sites. More research is warranted to better understand the circumstances under which epigenomics-based tests could be best utilized.

In the present study, we established genome-scale DNA methylation profiles of buffy coat samples from a nested case–control prospective study using reduced representation bisulphite sequencing (RRBS) to identify differentially methylated regions (DMRs) in breast cancer cases compared with controls. We observed that a Prediction Analysis for Microarrays (PAM) classification algorithm could discriminate individuals who developed breast cancer from those who did not. The final PAM model was tested on a held-out validation set, in which it was able to predict the occurrence of cancer in individuals months to years before clinical diagnosis of the disease.

## Results

### Study design

**Fig. 1:**
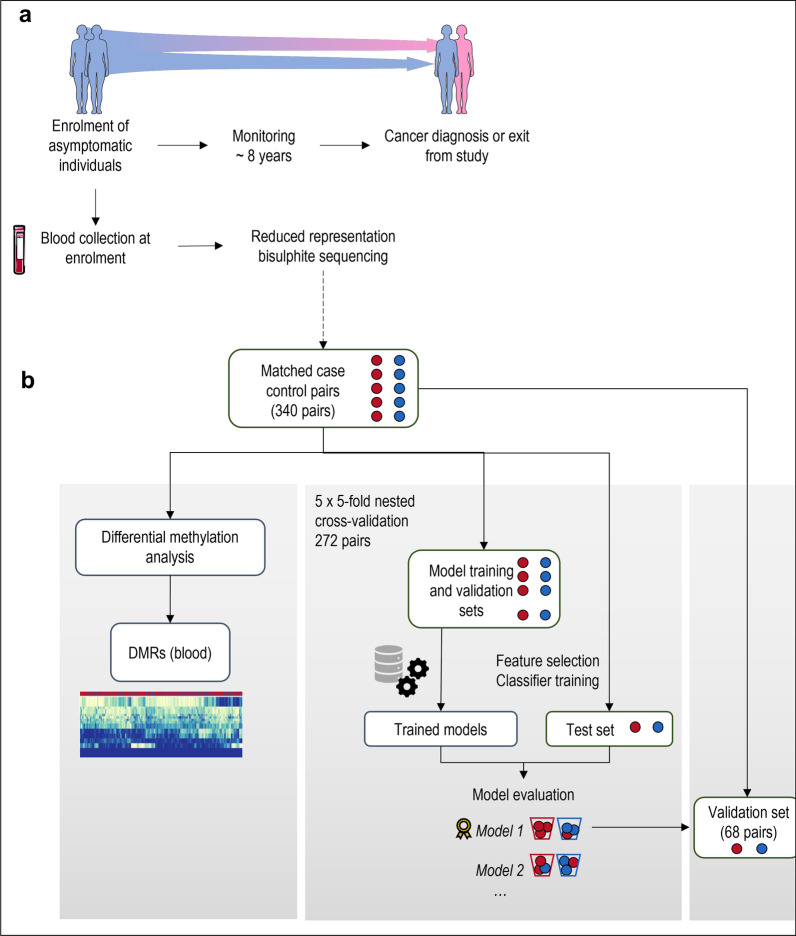
Schematic of the study design and analytical methods.** a** A nested case–control study was constructed within the EPIC-Heidelberg cohort. Blood samples and lifestyle information were collected from apparently healthy participants upon enrolment. Participants who were diagnosed with breast cancer over the course of follow-up were matched on a one-to-one basis with individuals who were observed to be cancer-free over the study period. Buffy coat lysates derived from blood samples collected at enrolment were analysed by reduced representation bisulphite sequencing (RRBS). **b** Fivefold nested cross-validation was used to train and evaluate classifier models for their ability to discriminate individuals who developed breast cancer from those who were cancer-free over the follow-up period. The best-performing model was selected and trained on the full cross-validation dataset of 340 pairs, to finalize model parameters. The final model was used to predict case–control status in a held-out validation set of 68 matched pairs. Differential methylation analyses and functional enrichment analyses were conducted in parallel. This figure uses images from Servier Medical Art licensed under a Creative Commons Attribution 3.0 Unported License (https://creativecommons.org/licenses/by/3.0/)

Samples from the EPIC-Heidelberg cohort, a sub-cohort of the European Prospective Investigation into Cancer and Nutrition (EPIC), study were used to construct a nested case–control study (study design is illustrated in Fig. 1). Blood samples were collected at enrolment from apparently healthy participants, from which buffy coat fractions were processed to yield a dataset of 702 RRBS profiles from 696 individuals. The final dataset consisted of 340 matched case–control pairs. Cohort characteristics are described in Additional file 1: Tables S1 and S2.

For predictive model development, 272 randomly selected matched pairs (80%) constituted a primary set that was used for model development and evaluation, and a set of 68 pairs (20%) was held out as a model validation set. Baseline cohort characteristics of the model development and validation sets are listed in Additional file 1: Table S2 and the distributions are graphed in Additional file 2: Fig. S1.

### Differentially methylated regions detected in prospectively collected buffy coat samples

**Fig. 2:**
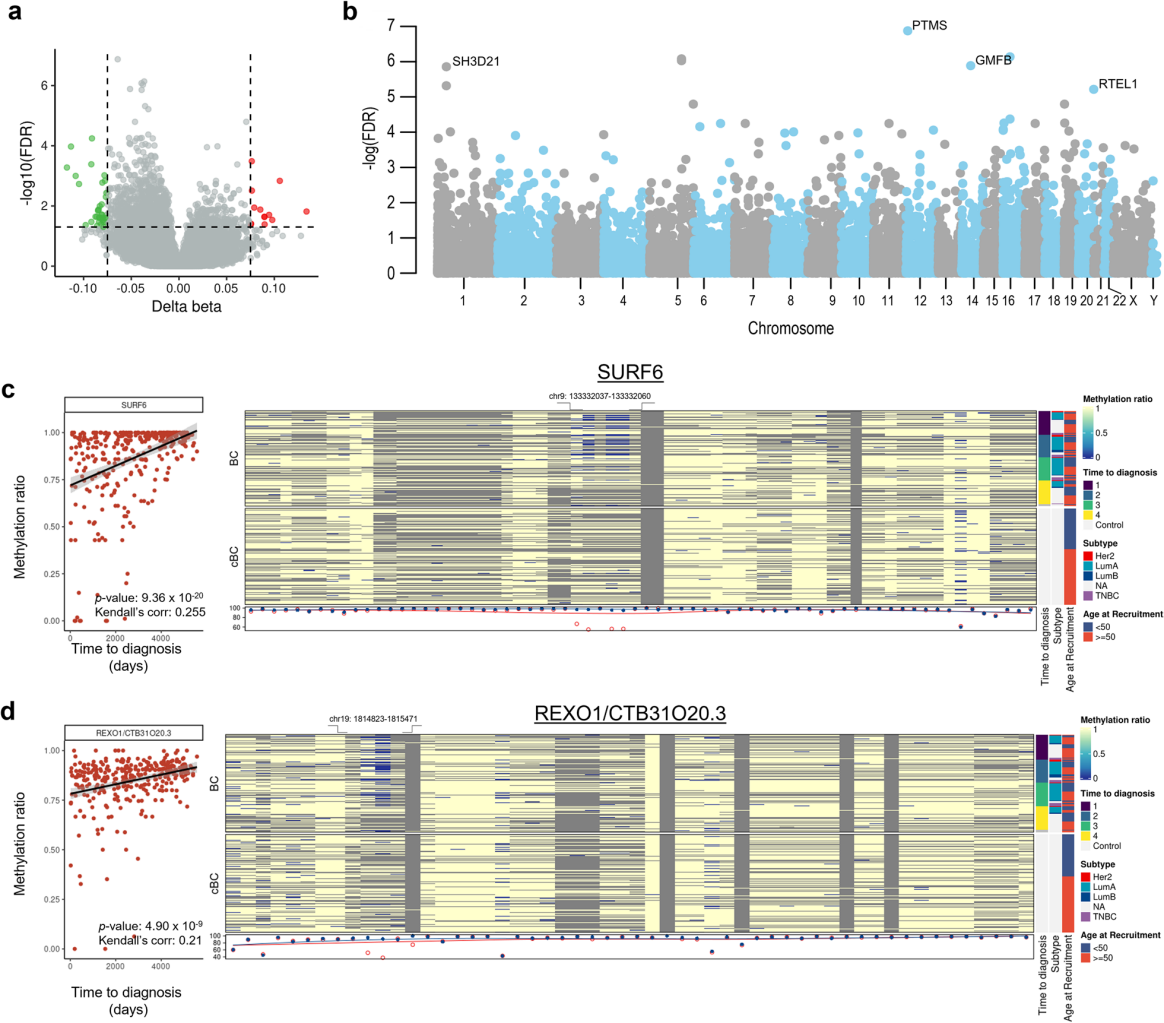
Differentially methylated sites detected in prospectively collected blood samples from individuals who develop cancer within the study timeframe when compared with age- and lifestyle-matched control individuals. **a** Volcano plot illustrating results of differential methylation analyses comparing regions annotated to promoters between case and control buffy coat samples. **b** Manhattan plot illustrating the chromosomal locations of the top differentially methylated sites between cases and controls. Scatterplots demonstrating a positive correlation between DNA methylation levels at **c** SURF6 and **d** REXO1/CTB31O20.3 and length of time to diagnosis suggest that progressive demethylation at these sites could be indicative of early carcinogenesis. CpG sites that were found to be driving this relationship are highlighted in the regional heat map on the right

Paired differential analyses between cases and controls yielded 187 significantly differentially methylated genomic regions associated with 165 genes (false discovery rate [FDR]-adjusted *p* value < 0.05, absolute mean difference in beta values > 0.075). The full list of DMRs is given in Additional file 3: Table S3, and a representative volcano and Manhattan plot illustrating results of comparisons within gene promoter regions is shown in Figs. 2a and 2b, respectively. When differential methylation analysis was conducted on pairs representing women diagnosed at above 50 years of age (representing post-menopausal breast cancer), 154 DMRs were identified, corresponding to 128 known genes (Additional file 4: Table S4). Notably, 104 of these regions, corresponding to 65 known genes, overlapped with DMRs identified in the main analysis with all matched pairs.

This included hypomethylation in genomic regions associated with oestrogen-related receptor beta (ESRRB) and the F-box protein member FBOX38 (Fig. 2a). Notably, ESRRB is a nuclear receptor and transcription factor which binds to the oestrogen-related receptor response element and is a key regulator in the reprogramming of pluripotent stem cells [30, 31] and glucocorticoid receptor signalling [32], whereas F-box proteins are members of the ubiquitin-protein E3 ligase family that play an important role in cell cycle regulation [33]. Pathway enrichment analysis of the genes associated with the 187 DMRs identified from the case–control comparison indicated that there were no significantly enriched gene ontologies or pathways after correction for multiple testing (Additional file 5: Table S5). Similar analysis for overlapping genes of the main analysis (all case–control pairs) and the post-menopausal pairs revealed significant enrichment for the carbohydrate:proton symporter activity GO Molecular Function term (Additional file 6: Table S6). The hypermethylated and hypomethylated regions of the main analysis (Additional file 3: Table S3) were significantly depleted for FANTOM5 enhancer regions identified in the GM12878 lymphoblastoid cell line relative to the total dataset (Fisher’s exact test, *p* < 0.05, Additional file 7: Fig. S2a). Similarly, the DMRs were depleted for promoter regions and were enriched for 1 to 5 kb regions and exonic regions relative to the total dataset (Additional file 7: Fig. S2b).

Of the 187 DMRs, 75 were significantly correlated with time to diagnosis in breast cancer cases (FDR-adjusted *p* value < 0.05, Additional file 3: Table S3). Regions most significantly correlated with time to diagnosis include SURF6 (Fig. 2c) and REXO1/CTB31O20.3 (Fig. 2d, Additional file 8: Table S7). CpG sites within the regions chr9: 133,332,037–133,332,060 for SURF6 and chr19: 1,814,823–1,815,471 for REXO1 were lowly methylated in cases that were diagnosed within 21–2665 days after recruitment (i.e. within the first and second quartile of patients by time to diagnosis), whereas higher levels of methylation were detected in matched controls and cases diagnosed more than 2665 days after recruitment.

### Identification of a panel of epigenetic predictors for breast cancer risk in RRBS dataset

Several classifiers were tested for their ability to discriminate between cases and controls (a schematic of the approach is illustrated in Fig. 1b) using fivefold cross-validation. The PAM classifier was the best-performing classifier overall when evaluated based on area under the receiver operating characteristic (ROC) curve (AUC), accuracy, sensitivity, and specificity (Additional file 1: Table S8, Additional file 10: Fig. S3). The PAM model used 49 genomic regions, corresponding to 38 known or predicted genes (Additional file 9: Table S9).

**Fig. 3:**
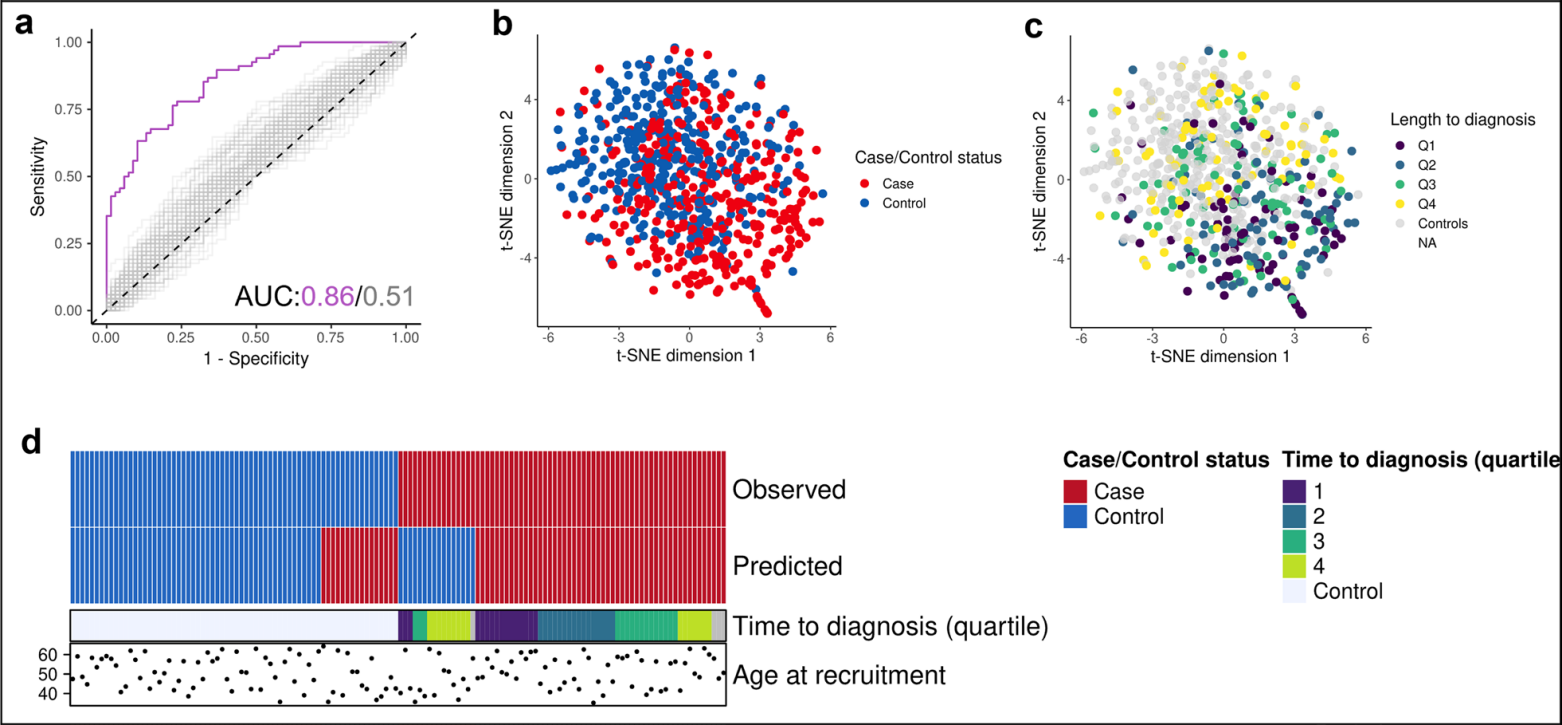
Prediction of case–control status in prospectively collected blood samples using a PAM classifier.** a** The receiver operating characteristic curve and the corresponding area under the curve (AUC) statistics for the PAM classifier applied on the validation cohort, against a background of 100 label-shuffled control datasets that were subjected to the same model training and testing process. A *t*-distributed stochastic neighbour embedding (*t*-SNE) plot was generated using the 49 genomic regions used in the PAM classifier, coloured by **b** case–control status and **c** length of time from sample collection to diagnosis (by quartile). **d** Schematic of the classification results from the final PAM model on the held-out validation set alongside length of time to diagnosis (quartiles)

The PAM model was used to predict case–control status in the held-out set of 68 case–control pairs that were not used at any point during model development. The classifier correctly predicted case–control status in 52 of 68 cases, corresponding to an accuracy of 76.5%. The corresponding ROC curve and AUC statistic are shown in Fig. 3a, against a background of 100 label-shuffled datasets subjected both to the same feature selection (RFE) and classifier training process. The 49 predictive genomic regions used in the PAM classifier were used to generate a *t-*distributed stochastic neighbour embedding (*t*-SNE) plot, which showed considerable overlap between the case and control clusters (Fig. 3b). The cases most distinct from the controls were derived primarily from participants in the first and second quartiles by time to diagnosis (Fig. 3c). 13 of the 16 misclassified samples were in the third or fourth quartile of duration from sample collection to diagnosis, suggesting that the time to diagnosis could be an important factor influencing the performance of the predictors (Fig. 3d).

## Discussion

Epigenetic mechanisms play an integral role in coordinating spatiotemporal gene expression, enabling the emergence of diverse cell type-specific phenotypes [20]. In cancers, one of the most well-described epigenetic aberrations is DNA hypomethylation within intergenic regions and/or partially methylated domains, punctuated by hypermethylation of CpG-dense regions [34–37].

Differentially methylated regions in the buffy coat samples include regions associated with surfeit locus protein 6 (SURF6), deregulation of which have been reported in the peripheral blood cells of breast cancer patients [38]; ESRRB, a key regulator of stem cell pluripotency [30] and self-renewal [39, 40]; and FBXO38, which mediates the ubiquitination and degradation of the substrate programmed cell death protein 1 (PD-1) [41]. We observed that DNA methylation levels in a subset of these DMRs were significantly associated with length of time to diagnosis, lending confidence to our hypothesis that gradual alterations in DNA methylation states indicative of early phases of tumour development are detectable in the blood (buffy coat) samples prior to clinical diagnosis. Similar observations were reported by Xu et al. (2020), whereby 42.6% of the CpG sites found to be differentially methylated between cases and controls were significantly correlated with time to diagnosis [24]. Here, the authors opined that this progressive divergence suggests that the detected alterations to blood DNA methylation are an early response to tumour development, rather than a long-term marker of breast cancer susceptibility, where in the latter case blood DNA methylation alterations would be expected to be independent of time to diagnosis.

Additionally, we postulate that as these epigenetic alterations were detected in buffy coats, epigenetic alterations in these regions may not necessarily reflect the molecular/cellular alterations leading to or arising from carcinogenesis in the target tissue, and could instead be reflections of molecular/cellular processes associated with the early stages of tumour development, such as chronic inflammation and accelerated ageing, deleterious exposures, or any combination of the above, the identification of which is beyond the scope of the current study. Additionally, the observed epigenetic alterations may also result from changes to the composition of cell types in the buffy coats analysed, a factor which is documented to be sensitive to chronic and acute stressors [42, 43]. While methodologies for deconvoluting cell-type composition have been well-established for array-based datasets, similar methodologies have yet to be used widely with RRBS datasets. Due to the fact that methodologies for deconvolution are not suited to be applied across platforms [44], cell-type deconvolution was not conducted in this study. Thus, the epigenetic alterations observed may have arisen due to alterations in cell-type composition, or due to large epigenetic alterations in specific cell types. Regardless, the processes resulting in the alterations observed in this study should be of a stable or persistent nature as the resulting epigenetic events persist through the continuous renewal of blood cells. Whether these alterations occur as a result of persistent “provoking conditions”, or stable epigenomic alterations in progenitor cells or in long-lived specialized blood cells (e.g. memory lymphocytes) [43] remains to be determined. Further studies are thus warranted to determine if the isolation of specific cell populations for DNA methylation analysis will prove beneficial for identifying robust biomarkers in prospectively collected blood samples [43].

While predictor performance reported in this study is lower than that in previous studies, we note that the current study uses prospectively collected blood samples, compared with the majority of current reports, in which analyses were conducted on samples from participants already diagnosed with the disease [20–22]. Moreover, the aforementioned studies utilized cell-free DNA isolated from plasma or serum as opposed to buffy coat samples.

This finding also follows two studies exploring the epigenetic differences between cases and controls in prospective studies, which reported contrasting results [24, 28]. We emphasize that these findings do not indicate that the use of circulating biomarkers could be applied in diagnosis of the disease, but could serve as an important component of personalized risk-based early prevention strategies [4]. Because it is accepted that breast cancer risk can be best predicted by a combination of parameters including age, genetic variants, mammographic breast density, reproductive history and lifestyle factors, the present study provides a novel epigenetic risk classifier and demonstrates the potential utility of DNA methylation markers in detecting early cellular alterations involved in tumour development. However, as genomic and mammographic screening information were not available in the context of this study, the performance of epigenomic predictors in conjunction with polygenic risk scores, family history, and other predictors of breast cancer risk are beyond the scope of this study. We also acknowledge that although we tested the performance of the PAM classifier on a held-out validation set, this does not preclude the need for replication on a larger, independent cohort. Because large-scale longitudinal studies entail significant costs and logistical challenges, similarly designed studies applying RRBS for DNA methylation analyses have been limited. However, progress in similarly designed prospective studies in recent years [26, 27] could present an opportunity for these challenges to be overcome in the near future.

Secondly, we acknowledge that analysis of high-dimensional omics-derived datasets by machine learning methods could be vulnerable to overfitting. To mitigate this risk, we included feature reduction steps within our analyses and used a nested cross-validation approach to train the classifier models in addition to evaluate their performance on a held-out validation set, against parallel analyses using label-shuffled datasets. As epigenome-wide analyses and machine learning algorithms improve and become more accessible, we could be poised to see the integration of epigenetic signatures in risk stratification and screening protocols, opening new horizons in the fields of diagnostics and risk prediction, and this could prove to play a critical role in overcoming the challenges of bringing a robust epigenetics-based risk prediction tool to the clinic.

## Conclusions

The findings of this study suggest that gradually accumulated DNA methylation patterns in peripheral blood may occur before clinical manifestation of cancer. Further studies of these changes may provide useful markers for risk stratification and, ultimately, personalized cancer prevention.

## Materials and methods

### Study cohort

The present analysis uses a nested case–control study design with samples from the EPIC-Heidelberg study. Detailed information is provided in the Additional file 1. RRBS was conducted on 739 blood samples collected from women who reported breast cancer over the follow-up period (*n* = 359) and cancer-free control participants (*n* = 380). Matched controls were selected from cancer-free individuals within the cohort and were matched to cases by age at recruitment (± 5 years, with the exception of one pair that had an age difference of 9.9 years), menopausal status, and reported use of hormone therapy and/or contraceptives. All study participants provided written informed consent, and ethical approval for the EPIC study was obtained from the institutional review boards of the International Agency for Research on Cancer and local participating centres.

### Reduced representation bisulphite sequencing (RRBS) and data processing

RRBS was performed as previously described [45], based on DNA extracted from buffy coat samples and FFPE tumour as well as adjacent normal samples. RRBS libraries were sequenced using Illumina HiSeq 2000/3000/4000 platforms in a 50-bp single-end configuration. RRBS data were processed as previously described [45], using a custom pipeline based on Pypiper (v0.6) (http://code.databio.org/pypiper/) and Looper (v0.6) (http://code.databio.org/looper/). Exploratory analyses were conducted using workflows implemented in RnBeads [46]. Data presented consist of samples that have passed all quality control steps.

### Differential DNA methylation analysis

Differential DNA methylation analyses were conducted for buffy coat samples separately using the output from RnBeads with a custom bioinformatics pipeline [47]. Differences in DNA methylation profiles between cases and controls were identified using a linear model as implemented in the R/Bioconductor package limma [48, 49], with paired analyses, to account for account for the paired structure of the matched case–control study [46]. Batch correction was conducted on *M*-values using surrogate variable analysis as previously described [50]. Models were further adjusted for sequencing lane and length of time to diagnosis. The Enrichr gene list enrichment analysis tool was used to query the GO Biological Process 2021, GO Cellular Component 2021, GO Molecular Function 2021, Reactome 2022, and KEGG 2021 databases for pathway enrichment analysis of the identified DMRs [51, 52]. The annotatr package [53] was used to map DMRs and all analysed regions to genomic contexts as defined in the TxDb.Hsapiens.UCSC.hg38.knownGene and org.Hs.eg.db packages. DMRs were converted to hg19 regions using the liftOver function in the rtracklayer package and mapped to enhancer regions identified in GM12878 through the FANTOM5 project in annotatr [54].

### Marker selection, classifier training and evaluation

Several machine learning classifiers were implemented on mean-centred data using the R package caret. Mean-centring within matched pairs was carried out to account for the paired structure of the matched case–control study. Each classifier was applied on a subset of DNA markers provided by a backward feature selection method (RFE). The predictive performance of each classifier considered was finally assessed by implementing a fivefold nested cross-validation (CV) over 80% of the samples. The overall best-performing machine learning classifier was tested using a held-out set of 68 matched pairs, which were not used in the cross-validation and model development stages.

Full descriptions of the methods are provided in the Additional file 1.

## Supplementary Information

**Additional file 1.** Supplementary Methods, Supplementary Tables and References.

**Additional file 2: Fig. S1**. Distribution of participants within the model development and held-out sample sets byage at recruitment,body mass index,exit age, and proportion-of-whole graphs illustrating the distribution of participants bytumour subtype,menopausal status at recruitment,hormonal contraceptive use,hormone therapy use, andpregnancy history.

**Additional file 3. Supplementary Table 3.** List of significantly differentially methylated regions (FDR < 0.05, group mean difference > 0.075) between cases and matched controls in prospectively-collected buffy-coat samples. The correlation between DNA methylation levels at these regions to time to diagnosis was determined by Kendall rank correlation.

**Additional file 4. Supplementary Table 4.** List of significantly differentially methylated regions (FDR < 0.05, group mean difference > 0.075) between cases and matched controls in prospectively-collected buffy-coat samples, limited to samples collected from women diagnosed after the age of 50.

**Additional file 5. Supplementary Table 5.** Top GO Biological Processes, GO Cellular Components, GO Molecular Functions, Reactome, and KEGG Pathways enriched from significantly differentially methylated regions (FDR < 0.05, group mean difference > 0.075) identified when comparing between cases and matched controls in prospectively-collected buffy-coat samples.

**Additional file 6. Supplementary Table 6.** Top GO Biological Processes, GO Cellular Components, GO Molecular Functions, Reactome, and KEGG Pathways enriched from significantly differentially methylated regions (FDR < 0.05, group mean difference > 0.075) identified when overlapping DMRs identified in the main analysis with DMRs identified when samples were limited to participants aged 50 and above at recruitment.

**Additional file 7: Fig. S2.** Relative proportions of hypermethylated, hypomethylated and all regions of the dataset when annotated by Enhancer status as annotated in the FANTOM5 enhancer atlas for the GM12878 human lymphoblastoid cell line; andgenic annotations.

**Additional file 8. Supplementary Table 7.** Genomic regions in which DNA methylation is significantly correlated to length of time to diagnosis (days) by Kendall rank correlation (FDR < 0.05).

**Additional file 9. Supplementary Table 9.** Genomic regions utilized by the PAM prediction model.

**Additional file 10: Fig. S3**. ROC curves for the tested classifiers. Individual ROC curves are shown for each cross-validation fold. SVM: support vector machines; PLR: penalized logistic regression; NNET: neural network; RF: random forests; LogitBoost: boosted logistic regressison; KNN: k-nearest neighbours; PAM: Prediction Analysis for Microarrays; RPART: classification and regression tree

## Data Availability

Data generated in this manuscript are available upon reasonable request from the corresponding authors to comply with the IARC and DKFZ institute ethics regulations to protect patient privacy. All requests will be promptly reviewed to verify if request is subject to any intellectual property or confidentiality obligations. Any data and materials that can be shared will be released subject to a Data Transfer Agreement.

## Abbreviations

AUC: Area under the receiver operating characteristic
CpG: Cytosine-guanine oligodeoxynucleotide
DMR: Differentially methylated region
DNA: Deoxyribonucleic acid
ENCODE: Encyclopedia of DNA elements
EPIC: European Prospective Investigation into Cancer and Nutrition
FDR: False discovery rate
PAM: Prediction analysis for microarrays
RFE: Recursive feature elimination
ROC: Receiver operating characteristic
RRBS: Reduced representation bisulphite sequencing
*t*-SNE: *t*-Distributed stochastic neighbour embedding
