# Supplementary material for: Buffy coat signatures of breast cancer risk in a prospective cohort study
Source: Clin Epigenetics. 2023 Jun 12;15:102. doi: 10.1186/s13148-023-01509-6 (PMC10262593; doi:10.1186/s13148-023-01509-6)
Supplement: Supplementary file 1 — Additional file 1. Supplementary Methods, Supplementary Tables and References. [file 13148_2023_1509_MOESM1_ESM.docx]

**Supplementary Methods**

*Study Cohort*

Samples from this study originate from patients recruited through the EPIC-Heidelberg study, which is a sub-cohort of EPIC, a multicentre prospective cohort study involving 23 centres in 10 European countries aimed at investigating the associations between diet, lifestyle, genetic and epigenetic factors, and various types of cancers (1, 2). The EPIC-Heidelberg study (*N* = 25,500 participants) includes a collection of blood samples collected upon enrolment from apparently healthy participants, as well as extensive questionnaire information on health and lifestyle, including nutrition, exogenous hormone use, and reproductive history. FFPE tissue blocks of primary breast tumours have also been systematically collected for all incident cases of breast cancer (*n* > 400), for which detailed information on pathology and tumour subtyping has been recorded.

RRBS methylome profiling was conducted on 739 blood samples collected from women who reported breast cancer over the follow-up period (*n* = 359) and cancer-free control participants (*n* = 380). Matched controls were selected from cancer-free individuals within the cohort and were matched to cases by age at recruitment (± 5 years, with the exception of one pair that had an age difference of 9.9 years), menopausal status, and reported use of hormone therapy and/or contraceptives. Samples that had not passed sample quality control steps, outliers, and case–control sets that were incidence-matched (in which late-developing cases were matched to serve as controls for relatively early-developing cases) were excluded (*n* = 59), yielding a final dataset of 340 matched pairs. All study participants provided written informed consent, and ethical approval for the EPIC study was obtained from the institutional review boards of the International Agency for Research on Cancer and local participating centres.

*Reduced representation bisulphite sequencing (RRBS)*

Epigenome-wide profiling was conducted by RRBS, for its robust performance and to overcome limitations in genome-wide coverage inherent to array-based assays (3). RRBS was performed as previously described (4). For most samples, 100 ng of genomic DNA was used. Methylated and unmethylated spike-in controls were added at a concentration of 0.1%, to enable assessments of bisulphite conversion efficiency. DNA was digested using the restriction enzyme MspI, followed by fragment end-repair, A-tailing, and adaptor ligation. Based on the qPCR-determined effective library quantities for each sample, samples were pooled and subjected to bisulphite conversion, followed by library enrichment by PCR. Fragment size distributions were confirmed on Bioanalyzer High Sensitivity DNA chips (Agilent). RRBS libraries were sequenced by the Biomedical Sequencing Facility at CeMM and the DKFZ Genomics and Proteomics Core Facility using the Illumina HiSeq 2000/3000/4000 platform and the 50-bp single-end configuration.

*RRBS data processing*

RRBS data were processed as previously described (4), using a custom pipeline based on Pypiper (v0.6) (<http://code.databio.org/pypiper/>) and Looper (v0.6) (<http://code.databio.org/looper/>). Briefly, adapter sequences were trimmed using Trimmomatic (v0.32) and trimmed reads were aligned to the human reference genome (GRCh38) using BSMAP (v2.90) in RRBS mode (5, 6). DNA methylation calling was performed with a custom Python script (biseqMethCalling.py) as described previously (7). To assess bisulphite conversion efficiency, unmapped reads were aligned to the spike-in reference sequences using Bismark (v0.12.2), and DNA methylation calls for methylated and unmethylated controls were extracted. CpGs in repetitive regions according to the UCSC RepeatMasker track were excluded from further analysis. DNA methylation was analysed at single-CpG resolution and in binned format with mean DNA methylation values calculated across CpG islands, GENCODE promoter regions (1 kilobase upstream to 500 bases downstream of the annotated transcription start site), and gene bodies. Regions for which data were missing in more than 5% of samples were excluded from all samples, with the remaining missing data replaced by the within-group mean of the region in question. Samples with less than 1 million unique CpG site coverage were excluded. The dataset after filtering that was used for analysis consisted of 70,020 regions. Exploratory analyses were conducted using workflows implemented in RnBeads (8). Data presented consist of samples that have passed all quality control steps.

*Differential DNA methylation analysis*

Differential DNA methylation analyses were conducted for buffy coat samples using the output from RnBeads with a custom bioinformatics pipeline (<https://github.com/IARCbioinfo/methylkey/blob/dev/methylkey.nf>) that was built in-house using Nextflow (9). Briefly, methylation ratios were logit transformed to *M*-values and corrected for batch effects using surrogate variable analysis as implemented in the R/Bioconductor package sva and as previously described (10), protecting for case–control status. Differences in DNA methylation profiles between cases and controls were identified using a linear model as implemented in the R/Bioconductor package limma (11, 12), with paired analyses, to account for account for the paired structure of the matched case-control study (8). The models were adjusted for sequencing lane and length of time to diagnosis, which were identified as potential confounders by principal component analysis using the methylkey custom bioinformatics pipeline described above. Regions were considered significantly differentially methylated when between-group comparisons yielded FDR-adjusted *p*-values of < 0.05 and absolute mean pairwise difference of > 0.075 in the buffy coat samples, respectively.

*Marker selection, classifier training and evaluation*

We implemented several well-established machine learning classification methods (hereafter referred to as classifiers) on the mean-centred data, including radial support vector machines (SVM), L2 penalized logistic regression (PLR) along with stepwise variable selection, boosted logistic regression (LogitBoost), random forests (RF), neural network (NNET), Prediction Analysis for Microarrays (PAM), k-nearest neighbours (kNN), and recursive partitioning models (RPART) using the R package caret. Mean-centring within matched pairs was carried out to account for the paired structure of the matched case–control study. This approach was proposed as a means of making matched designs amenable to linear or nonlinear classification algorithms, which do not typically account for matched designs.

Each classifier was applied on a subset of DNA markers provided by a backward feature selection method (RFE), to reduce the number of highly correlated features and/or potentially uninformative markers. The predictive performance of each classifier considered was finally assessed by implementing a five-fold nested cross-validation (CV) that involved both the RFE and classification steps, considering several performance measures, including accuracy (Acc), area under the ROC curve (AUC), sensitivity (Se), specificity (Sp), and Cohen kappa over 80% of the samples. Acc is defined as the proportion of correctly classified samples, Se as the proportion of predicted cases among observed breast cancer cases, and Sp as the proportion of predicted controls among observed cancer-free controls. The ROC curve (AUC) is defined by the area of the curve plotting the Se against 1 – Sp for varying risk thresholds and provides a way to compare the separability for all possible risk thresholds. Fold assignments were performed in a stratified manner to ensure that the matched-pair data structure was preserved. The best-performing machine learning classifier – based on the AUC, Ac, Se, and Sp – was then used to predict for case–control status in a held-out set of 68 matched pairs (20% of the dataset), which were not used in the cross-validation and model development stages. Furthermore, to demonstrate the robustness against overfitting of the classification results obtained, the entire procedure (the RFE and PAM classifier training) – using the same tuning parameters – was repeated 100 independent times with random shuffling of the case–control assignments at the beginning of each analysis. Shuffling was done within matched pairs to ensure that the matched-pair data structure was still preserved in each iteration.

*Statistical analysis*

All *t*-SNE plots were constructed using the R package Rtsne (v0.15) with 1000 iterations and a perplexity of 50. Heat maps were created using the R package ComplexHeatmap (v2.0.0) using Euclidean and Kendall correlation distance as dissimilarity measures (13). ROCs and AUCs were computed with the R package pROC (v1.16.1) (14). Classifiers were implemented using the R package caret (v6.0-86). Figures were plotted using the R packages ggbio (v1.32.0) and ggplot2 (v3.3.2). Correlation between methylation levels and time to diagnosis was evaluated using the Kendall correlation test. All statistical methods were implemented using R statistical software (v3.6.0).

*Data availability*

Data generated in this manuscript are available upon reasonable request from the corresponding authors to comply with the IARC and DKFZ institute ethics regulations to protect patient privacy. All requests will be promptly reviewed to verify if request is subject to any intellectual property or confidentiality obligations. Any data and materials that can be shared will be released subject to a Data Transfer Agreement.

**Supplementary Table 1.** Characteristics of the study population from which buffy coat samples were collected and analysed.

|  | Buffy coat samples | |
| --- | --- | --- |
| Characteristics | Controls  (*n*=340) | Cases  (*n*=340) |
| Body mass index (kg/m^2^) | 24 (22–28) | 25 (22–27) |
| Age at recruitment (years) | 51.88 (44.72–57.92) | 51.95 (44.86–57.94) |
| Age at diagnosis (years) | – | 59.32 (52.30–64.06) |
| Time to diagnosis (days) | – | 2665 (1420–4224) |
| Follow-up period for controls (days) | 4791 (4408–5096) | – |
| Tumour stage at diagnosis  (EPIC classification) |  |  |
| *In situ* | – | 4 (1.18%) |
| Localised | – | 149 (43.82%) |
| Regional metastasis | – | 94 (27.65%) |
| Distal metastasis | – | 8 (2.35%) |
| Unknown | – | 85 (25.00%) |
| Menopausal status |  |  |
| Premenopausal | 111 (32.65%) | 111 (32.65%) |
| Postmenopausal | 173 (50.88%) | 172 (50.59%) |
| Perimenopausal | 51 (15%) | 50 (14.71%) |
| Surgical postmenopausal | 5 (1.47%) | 7 (2.06%) |
| Ever pregnant |  |  |
| No | 49 (14.41%) | 53 (15.59%) |
| Yes | 291 (85.59%) | 287 (84.41%) |
| Hormone therapy use |  |  |
| No | 197 (57.94%) | 195 (57.35%) |
| Yes | 143 (42.06%) | 145 (42.65%) |

* Table excludes samples which were not included in downstream analyses due to technical inadequacies. Continuous variables are presented as median (interquartile range, IQR), categorical variables are presented as *n* (%).

**Supplementary Table 2.** Characteristics of the study population from which buffy coat samples were collected and analysed that constituted the primary model development set and the validation set.

|  | Model development set | | Validation set | |
| --- | --- | --- | --- | --- |
| Characteristics | Controls  (*n*=272) | Cases  (*n*=272) | Controls  (*n*=68) | Cases (*n*=68) |
| Body mass index (kg/m^2^) | 24 (22–28) | 25 (22–27) | 24 (21–27) | 24 (22–27) |
| Age at recruitment (years) | 52.19 (45.44–57.85) | 52.08 (45.08–57.87) | 50.85 (42.82–58.01) | 50.76 (42.56–57.98) |
| Age at diagnosis (years) | – | 59.36 (53.17–64.26) | – | 56.78 (49.09–63.45) |
| Time to diagnosis (days) | – | 2773 (1471–4252) | – | 2458 (1188–3938) |
| Follow-up period for controls (days) | 4794 (4409–5093) | – | 4724 (4399–5169) | – |
| Tumour stage at diagnosis  (EPIC classification) |  |  |  |  |
| *In situ* | – | 1 (0.37%) | – | 3 (4.41%) |
| Localised | – | 117 (43.01%) | – | 32 (47.06%) |
| Regional metastasis | – | 77 (28.31%) | – | 17 (25.00%) |
| Distal metastasis | – | 6 (2.21%) | – | 2 (2.94%) |
| Unknown | – | 71 (26.10%) | – | 14 (20.59%) |
| Menopausal status |  |  |  |  |
| Premenopausal | 85 (31.25%) | 84 (30.88%) | 26 (38.24%) | 27 (39.71%) |
| Postmenopausal | 140 (51.47%) | 138 (50.74%) | 33 (48.53%) | 34 (50.00%) |
| Perimenopausal | 43 (15.81%) | 44 (16.18%) | 8 (11.76%) | 6 (8.82%) |
| Surgical postmenopausal | 4 (1.47%) | 6 (2.21%) | 1 (1.47%) | 1 (1.47%) |
| Ever pregnant |  |  |  |  |
| No | 36 (13.24%) | 43 (15.81%) | 13 (19.12%) | 10 (14.71%) |
| Yes | 236 (86.76%) | 229 (84.19%) | 55 (80.88%) | 58 (85.29%) |
| Hormone therapy use |  |  |  |  |
| No | 164 (60.29%) | 162 (59.56%) | 33 (48.53%) | 33 (48.53%) |
| Yes | 108 (39.71%) | 110 (40.44%) | 35 (51.47%) | 35 (51.47%) |

* Table excludes samples which were not included in downstream analyses due to technical inadequacies. Continuous variables are presented as median (IQR), categorical variables are presented as *n* (%).

**Supplementary Table 8.** Predictive performance of classifier algorithms assessed by five-fold cross validation. The performance metrics are expressed as mean values (standard deviation in parentheses) over the five cross-validation folds.

| **Algorithm** | **AUC** | **Accuracy** | **Sensitivity** | **Specificity** | **Kappa** |
| --- | --- | --- | --- | --- | --- |
| PAM | 0.762 (0.056) | 0.68 (0.055) | 0.680 (0.055) | 0.680 (0.055) | 0.359 (0.11) |
| RF | 0.757 (0.032) | 0.685 (0.034) | 0.702 (0.035) | 0.669 (0.035) | 0.370 (0.067) |
| RPART | 0.753 (0.053) | 0.662 (0.033) | 0.647 (0.022) | 0.677 (0.058) | 0.324 (0.066) |
| KNN | 0.719 (0.059) | 0.66 (0.054) | 0.655 (0.059) | 0.664 (0.058) | 0.319 (0.109) |
| NNET | 0.712 (0.041) | 0.675 (0.059) | 0.677 (0.055) | 0.673 (0.062) | 0.350 (0.117) |
| LogitBoost | 0.702 (0.062) | 0.634 (0.083) | 0.622 (0.086) | 0.647 (0.085) | 0.268 (0.167) |
| PLR | 0.692 (0.043) | 0.652 (0.04) | 0.632 (0.064) | 0.673 (0.03) | 0.305 (0.079) |
| SVM | 0.69 (0.084) | 0.632 (0.077) | 0.632 (0.077) | 0.632 (0.077) | 0.264 (0.153) |

PAM: Prediction Analysis for Microarrays; RF: random forests; RPART: classification and regression tree; KNN: k-Nearest neighbour; PLR: penalized logistic regression; LogitBoost: boosted logistic regression; NNET: neural network; SVM: support vector machines.

**Supplementary References**

1. Riboli E, Hunt KJ, Slimani N, Ferrari P, Norat T, Fahey M, et al. European Prospective Investigation into Cancer and Nutrition (EPIC): study populations and data collection. Public Health Nutrition. 2007;5(6b):1113-24.

2. Riboli E, Kaaks R. The EPIC Project: rationale and study design. European Prospective Investigation into Cancer and Nutrition. International Journal of Epidemiology. 1997;26(suppl_1):S6-S.

3. Carmona JJ, Accomando WP, Jr., Binder AM, Hutchinson JN, Pantano L, Izzi B, et al. Empirical comparison of reduced representation bisulfite sequencing and Infinium BeadChip reproducibility and coverage of DNA methylation in humans. NPJ Genom Med. 2017;2:13-.

4. Klughammer J, Kiesel B, Roetzer T, Fortelny N, Nemc A, Nenning K-H, et al. The DNA methylation landscape of glioblastoma disease progression shows extensive heterogeneity in time and space. Nat Med. 2018;24(10):1611-24.

5. Xi Y, Li W. BSMAP: whole genome bisulfite sequence MAPping program. BMC Bioinformatics. 2009;10:232-.

6. Xi Y, Bock C, Müller F, Sun D, Meissner A, Li W. RRBSMAP: a fast, accurate and user-friendly alignment tool for reduced representation bisulfite sequencing. Bioinformatics. 2012;28(3):430-2.

7. Klughammer J, Datlinger P, Printz D, Sheffield NC, Farlik M, Hadler J, et al. Differential DNA Methylation Analysis without a Reference Genome. Cell Rep. 2015;13(11):2621-33.

8. Müller F, Scherer M, Assenov Y, Lutsik P, Walter J, Lengauer T, et al. RnBeads 2.0: comprehensive analysis of DNA methylation data. Genome Biology. 2019;20(1):55.

9. Di Tommaso P, Chatzou M, Floden EW, Barja PP, Palumbo E, Notredame C. Nextflow enables reproducible computational workflows. Nature Biotechnology. 2017;35(4):316-9.

10. Perrier F, Novoloaca A, Ambatipudi S, Baglietto L, Ghantous A, Perduca V, et al. Identifying and correcting epigenetics measurements for systematic sources of variation. Clinical Epigenetics. 2018;10(1):38.

11. Phipson B, Lee S, Majewski IJ, Alexander WS, Smyth GK. Robust hyperparameter estimation protects against hypervariable genes and improves power to detect differential expression. The annals of applied statistics. 2016;10(2):946-63.

12. Ritchie ME, Phipson B, Wu D, Hu Y, Law CW, Shi W, et al. limma powers differential expression analyses for RNA-sequencing and microarray studies. Nucleic acids research. 2015;43(7):e47.

13. Kuleshov MV, Jones MR, Rouillard AD, Fernandez NF, Duan Q, Wang Z, et al. Enrichr: a comprehensive gene set enrichment analysis web server 2016 update. Nucleic acids research. 2016;44(W1):W90-7.

14. Robin X, Turck N, Hainard A, Tiberti N, Lisacek F, Sanchez J-C, et al. pROC: an open-source package for R and S+ to analyze and compare ROC curves. BMC Bioinformatics. 2011;12(1):77.
