# Supplementary figures and images for: Buffy coat signatures of breast cancer risk in a prospective cohort study
Source: Clin Epigenetics. 2023 Jun 12;15:102. doi: 10.1186/s13148-023-01509-6 (PMC10262593; doi:10.1186/s13148-023-01509-6)

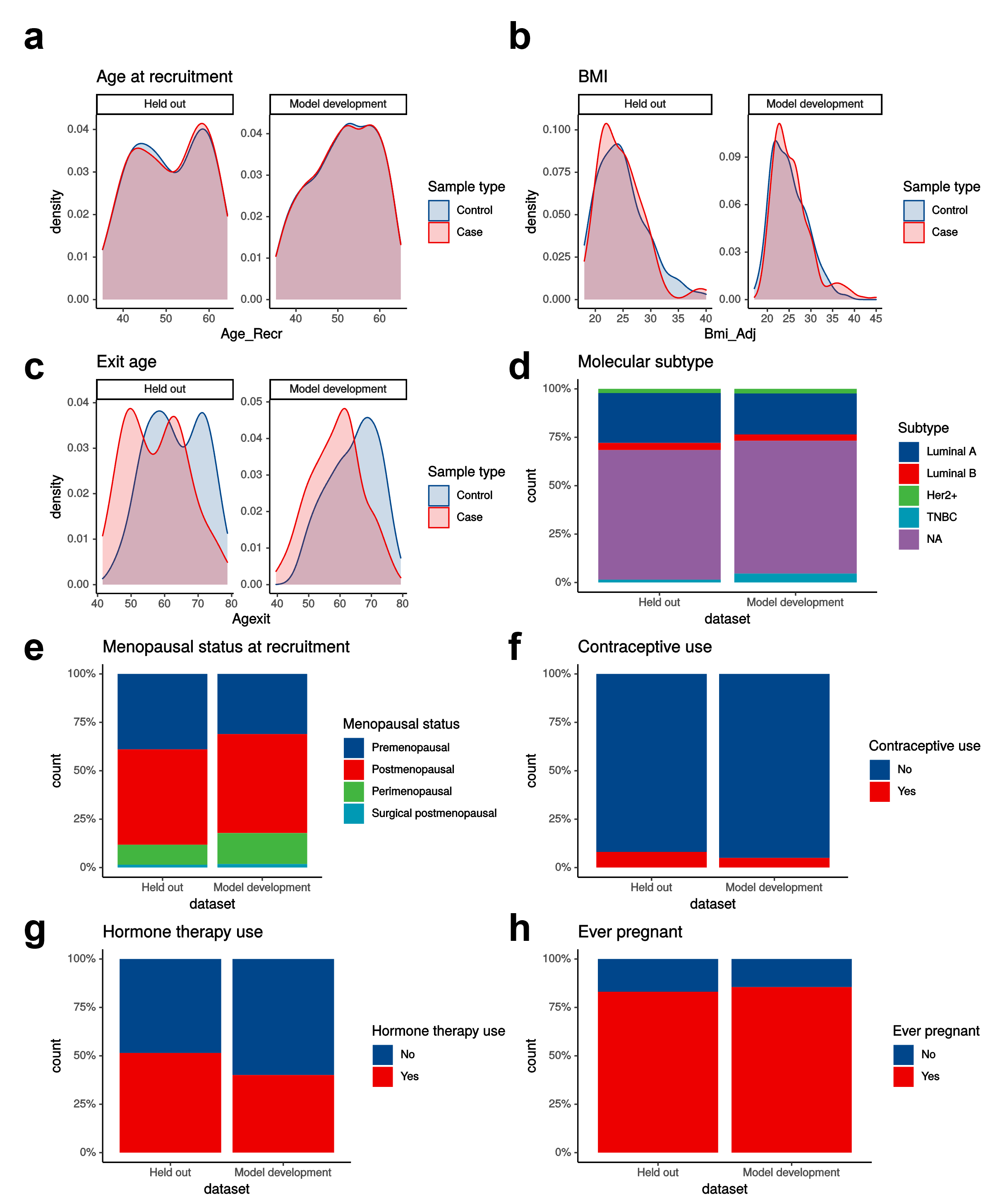

Supplement: Supplementary file 2 — Additional file 2: Fig. S1. Distribution of participants within the model development and held-out sample sets byage at recruitment,body mass index,exit age, and proportion-of-whole graphs illustrating the distribution of participants bytumour subtype,menopausal status at recruitment,hormonal contraceptive use,hormone therapy use, andpregnancy history. [file 13148_2023_1509_MOESM2_ESM.tif]

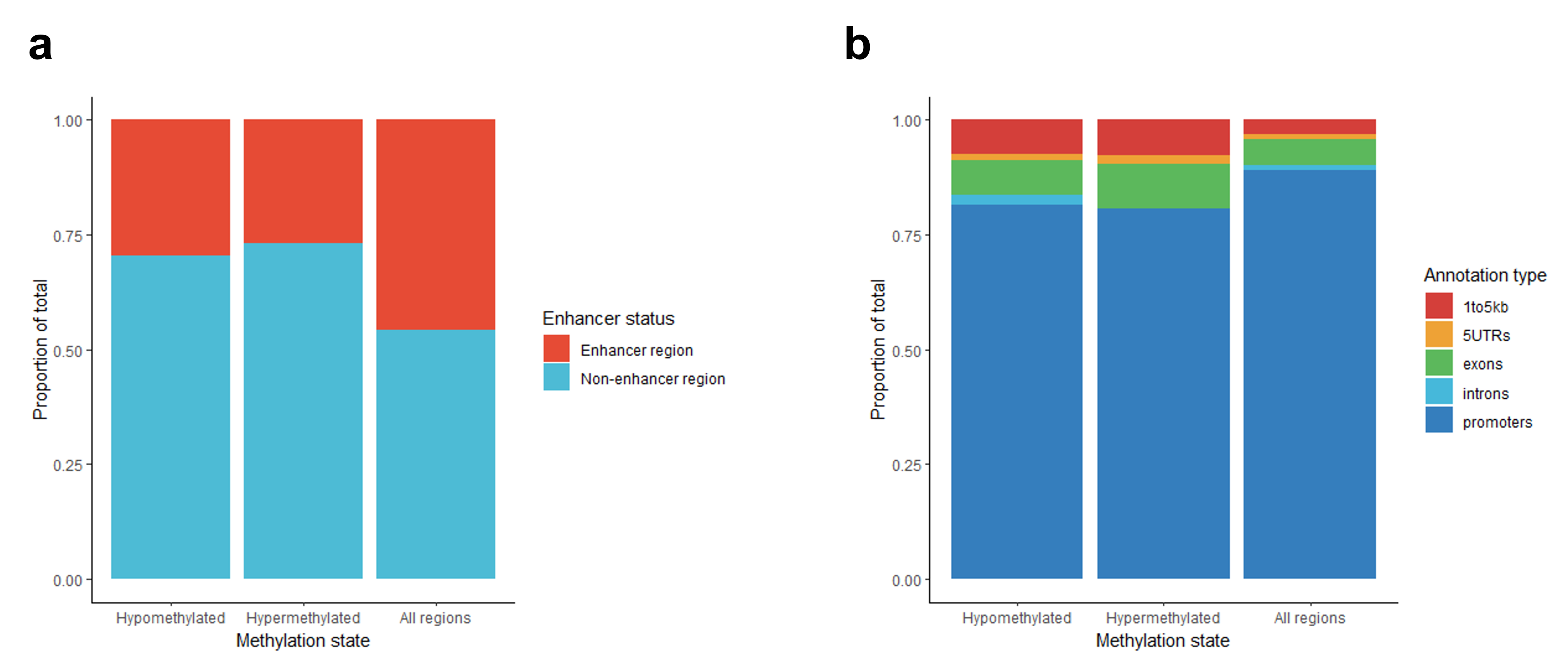

Supplement: Supplementary file 7 — Additional file 7: Fig. S2. Relative proportions of hypermethylated, hypomethylated and all regions of the dataset when annotated by Enhancer status as annotated in the FANTOM5 enhancer atlas for the GM12878 human lymphoblastoid cell line; andgenic annotations. [file 13148_2023_1509_MOESM7_ESM.tif]

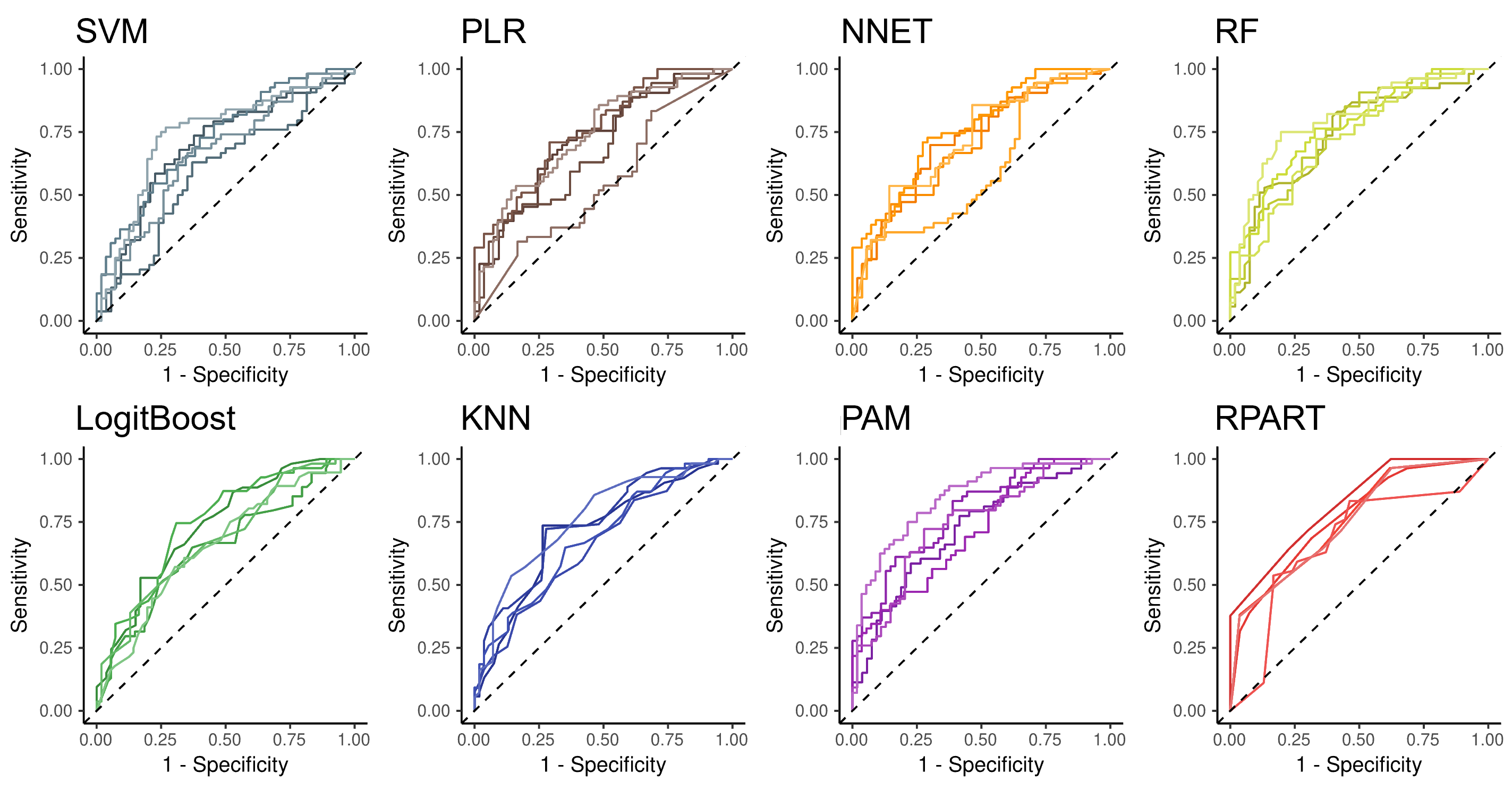

Supplement: Supplementary file 10 — Additional file 10: Fig. S3. ROC curves for the tested classifiers. Individual ROC curves are shown for each cross-validation fold. SVM: support vector machines; PLR: penalized logistic regression; NNET: neural network; RF: random forests; LogitBoost: boosted logistic regressison; KNN: k-nearest neighbours; PAM: Prediction Analysis for Microarrays; RPART: classification and regression tree [file 13148_2023_1509_MOESM10_ESM.tif]
